# Protein palmitoylation: biological functions, disease, and therapeutic targets

**DOI:** 10.1002/mco2.70096

**Published:** 2025-02-21

**Authors:** Yan‐Ran Qian, Yu‐Jia Zhao, Feng Zhang

**Affiliations:** ^1^ Key Laboratory of Basic Pharmacology of Ministry of Education and Joint International Research Laboratory of Ethnomedicine of Ministry of Education and Key Laboratory of Basic Pharmacology of Guizhou Province and Laboratory Animal Centre Zunyi Medical University Zunyi Guizhou China

**Keywords:** biological functions, neurological disorders, palmitoylation, post‐translational modifications, therapeutic targets

## Abstract

Protein palmitoylation, a reversible post‐translational lipid modification, is catalyzed by the ZDHHC family of palmitoyltransferases and reversed by several acyl protein thioesterases, regulating protein localization, accumulation, secretion, and function. Neurological disorders encompass a spectrum of diseases that affect both the central and peripheral nervous system. Recently, accumulating studies have revealed that pathological protein associated with neurological diseases, such as β‐amyloid, α‐synuclein, and Huntingtin, could undergo palmitoylation, highlighting the crucial roles of protein palmitoylation in the onset and development of neurological diseases. However, few preclinical studies and clinical trials focus on the interventional strategies that target protein palmitoylation. Here, we comprehensively reviewed the emerging evidence on the role of protein palmitoylation in various neurological diseases and summarized the classification, processes, and functions of protein palmitoylation, highlighting its impact on protein stability, membrane localization, protein–protein interaction, as well as signal transduction. Furthermore, we also discussed the potential interventional strategies targeting ZDHHC proteins and elucidated their underlying pathogenic mechanisms in neurological diseases. Overall, an in‐depth understanding of the functions and significances of protein palmitoylation provide new avenues for investigating the mechanisms and therapeutic approaches for neurological disorders.

## Graphical abstract

This review focuses on the role of protein palmitoylation in neurological disorders, with an emphasis on biological function and therapeutic targets. ZDHHC protein mediated palmitoylation is strongly implicated in epilepsy, MDD, ID, and SCZ, and in addition, enzymes that mediate palmitoylation may be novel targets in the treatment of  neurological diseases.

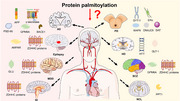

## 1 INTRODUCTION

**FIGURE 1:**
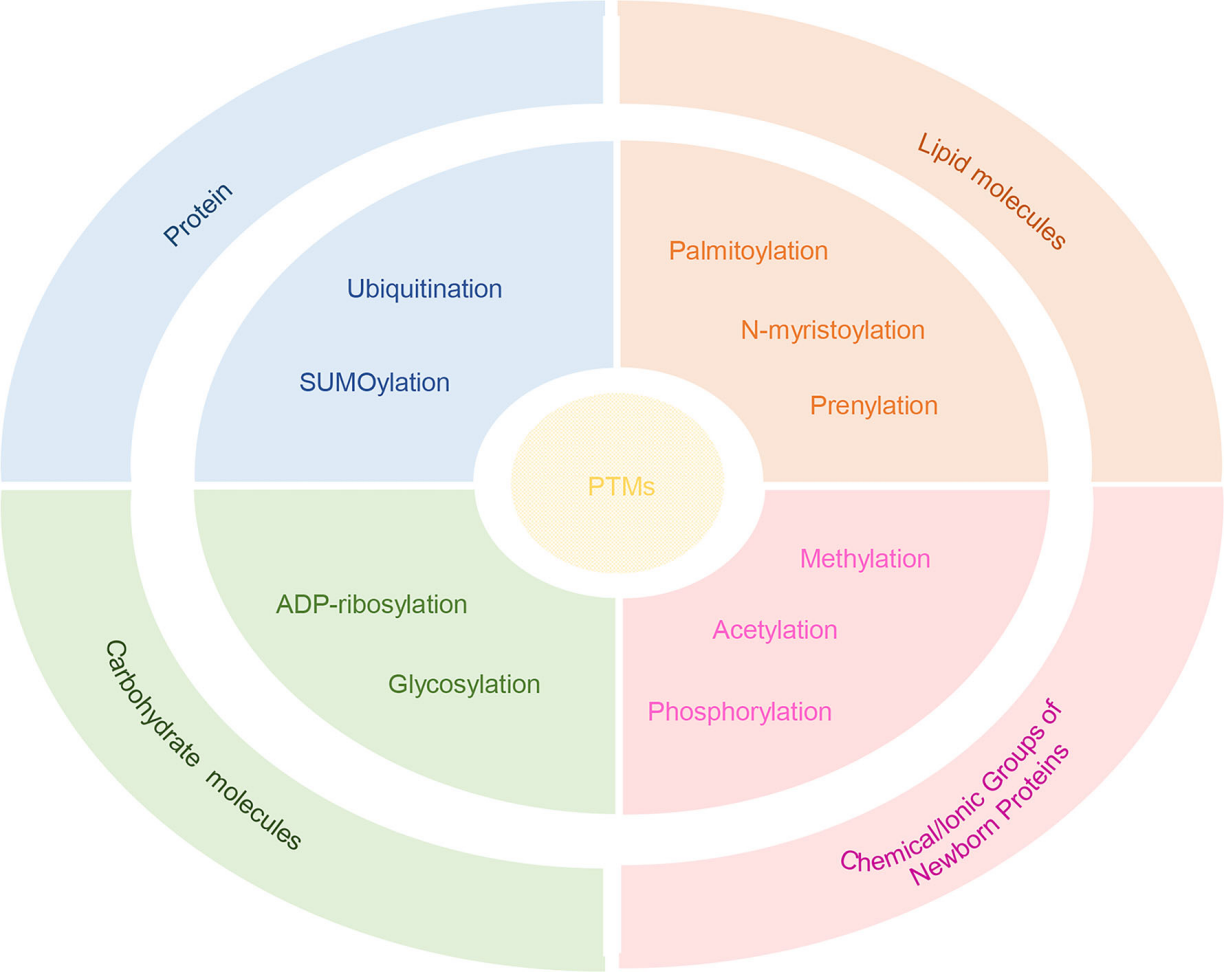
Classification of PTMs. Protein‐based modifications were classified as ubiquitination and SUMOylation; carbohydrate molecule‐based modifications were classified as glycosylation and ADP‐ribosylation; lipid molecule‐based modifications were classified as palmitoylation, N‐myristoylation, and prenylation; and chemical/ionic groups based of newborn proteins were classified as acetylation, phosphorylation, and methylation. PTMs, post‐translational modifications.

Protein post‐translational modifications (PTMs), the main mechanisms for regulating protein function, are essential for cellular regulation and signal transduction.^1^, ^2^ Specific enzymes, including ubiquitin E3 ligases, glycosyltransferases, poly adenosine diphosphate ribose polymerase, acetyltransferases, and kinases, are responsible for carrying out these processes.^3^, ^4^ Currently, PTMs can be widely categorized into four categories: (1) protein‐based modifications involving ubiquitin (ubiquitination), SUMO (SUMOylation), ISG (ISGylation), and NEDD8 (NEDDylation); (2) modifications based on carbohydrate molecules, such as glycosylation, ADP‐ribosylation; (3) modifications based on lipid molecules (palmitoylation, N‐myristoylation, prenylation); and (4) modifications involving chemical/ionic groups of nascent proteins, such as acetyl, phosphoryl, and methyl groups (Figure 1).^4^ In recent years, palmitoylation based on lipid molecular modifications has gradually gained attention in the scientific community.

Palmitoylation was initially documented in 1979.^5^ Palmitoylation, the most‐studied protein lipidation modification, encompasses the reversible covalent attachment of palmitate moieties to protein cysteine (Cys) residues.^6^ Evidence indicated that palmitoylation is predominantly observed in neurons, suggesting its potential relevance to the development of neurological diseases.^7^, ^8^ This process is mainly mediated by palmitoyl acyltransferases (PAT), also known as ZDHHC enzymes, which are responsible for the palmitoylation of protein.^9^ Majority of ZDHHC enzymes are predominantly found in the endoplasmic reticulum (ER) and Golgi apparatus, although a minority is also present in the plasma membrane and endosomes.^10^, ^11^, ^12^ Increasing studies suggest that these enzymes play critical roles in neuronal development and function. Moreover, enzymes, acyl protein thioesterases (APT) or palmitoyl protein thioesterase (PPT), are involved in protein depalmitoylation.^13^ Increasing evidence supports that palmitoylation plays a crucial role in the pathogenesis of neurological disorders, such as Alzheimer's disease (AD), Parkinson's disease (PD), Huntington's disease (HD), Schizophrenia (SCZ), Epilepsy, Intellectual disability (ID), Major depressive disorder (MDD), and Neuronal ceroid lipofuscinoses (NCL).^14^, ^15^, ^16^, ^17^, ^18^, ^19^, ^20^

Neurological disorders are a range of disorders that affect the structural integrity of the central and peripheral nervous system, resulting in various forms of damage and injury to the brain, spinal cord, cranial and peripheral nerve set, among others.^21^ As an example, protein palmitoylation enhanced the accumulation of pathogenic proteins associated neurological disorders, such as β‐amyloid (Aβ), α‐synuclein (α‐syn), and Huntingtin (HTT).^15^, ^22^ In addition, palmitoylation increased the risk of SCZ.^23^ In epilepsy, ID, MDD, and NCL, the palmitoylation‐associated protein ZDHHCs or enzymes involved in palmitoylation/depalmitoylation are significantly associated with the onset and progression of these disorders.^24^, ^25^, ^26^, ^27^ Taken together, protein palmitoylation is common phenomenon in various neurological disorders.

In this review, we provided comprehensive evidence on the relationship between protein palmitoylation and neurological diseases, summarized its classification, processes, and functions, with an emphasis on aspects such as protein stability, membrane localization, protein–protein interactions, and signal transduction. In addition, we discussed potential intervention strategies for targeting ZDHHC proteins and elucidated their potential pathogenic mechanisms in neurological diseases. Overall, understanding the role and importance of protein palmitoylation opens novel pathways for exploring mechanisms and therapeutic strategies in neurological disorders.

## 2 PROTEIN PALMITOYLATION: CLASSIFICATION, PROCESS, AND FUNCTIONS

### 2.1 Classification and process

It involved the covalent attachment of long‐chain fatty acids to protein Cys sites, including S‐palmitoylation,^26^ N‐palmitoylation,^27^ an O‐palmitoylation.^28^ N‐palmitoylation refers to the covalent attachment of a palmitoyl group to the amino group of glycine, lysine, or Cys residues through the formation of a stable amide bond,^27^, ^29^ and this modification was not reversible. Currently, the repertoire of proteins undergoing N‐palmitoylation is comparatively restricted relative to other palmitoylation forms. Conversely, O‐palmitoylation, which involves the esterification of serine residues,^28^ is an infrequent modification. Among them, S‐palmitoylation comprises the binding of saturated 16C palmitate to protein Cys sulfhydryl groups via thioester bonds. Under specific conditions, the thioester bond can undergo hydrolysis, leading to the dissociation of palmitate from the Cys sulfhydryl group.^30^ S‐palmitoylation is a critical mechanism for modulating protein function via influencing protein interactions with membranes, cellular compartments, transport processes, cellular localization, and stability.^31^ However, the “Swiss Palm” database shows that more than 10% of the entire human proteome is sensitive to S‐palmitoylation.^32^ Noteworthy, S‐palmitoylation is a reversible protein modification, whereas N‐palmitoylation and O‐palmitoylation are not.^33^, ^34^ This shows the importance of S‐palmitoylation in distinguishing it from the other two palmitoylation modifications.

**FIGURE 2:**
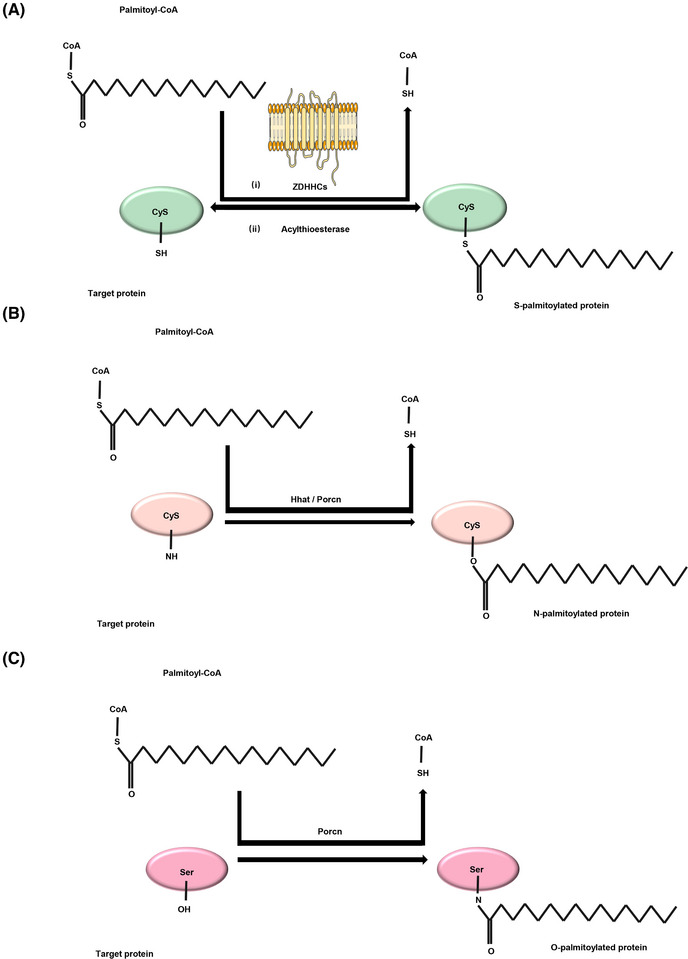
Palmitoylation modification process. S‐palmitoylation modification, N‐palmitoylation modification, and O‐palmitoylation modification process. (A) (i) An autoacylation reaction occurs in which palmitoyl CoA and ZDHHC motif form an acylase intermediate, during which free CoA‐SH is released. In the absence of a substrate, the intermediate undergoes slow hydrolysis, and (ii) in the presence of the acylthioesterase, the palmitoyl group of the intermediate bond is transferred to the target protein. (B) N‐palmitoylation is the attachment of a palmitoyl group to the amino group of a glycine, lysine, or cysteine residue via a stable amide bond. (C) O‐palmitoylation occurs in serine residues via ester bonds.

Protein S‐palmitoylation modification consists of two main processes (Figure 2). First, an auto‐acylation reaction occurs where palmitoyl Coenzyme（CoA） and the Cys of the palmitoyl group transfer enzyme form an acylase intermediate through a thioester bond, releasing free   CoA‐SH in this process. In the absence of a substrate, the intermediate undergoes slow hydrolysis. Second, under substrate‐containing conditions, the intermediate‐linked palmitoyl group is transferred to the substrate Cys.^34^

Palmitoylation is the most prevalent post‐translational lipid modification in the brain, with the identification of 24 mammalian ZDHHC proteins, most of which have exhibited PAT activity in both yeast^35^ and mammalian cells.^4^, ^36^ ZDHHC proteins, which are evolutionarily conserved across eukaryotes, are encoded by multigene families and responsible for the palmitoylation of numerous protein substrates.^37^ All ZDHHCs are integral membrane proteins containing at least 4 transmembrane structural domains. The catalytic ZDHHC (aspartate–histidine–histidine–Cys) Cys‐rich structural domain (CRD) was present on the cytoplasmic loop.^38^ Currently, more evidence indicated that PATs played critical roles in the pathogenesis of neurological diseases. Specifically, DHHC3, DHHC4, DHHC7, DHHC15, and DHHC20 had been identified as PAT responsible for the palmitoylation of β‐site amyloid precursor protein shear (APP) enzyme 1 (BACE1), a gene involved in the progression of AD.^39^ In addition, ZDHHC8, ZDHHC5, and ZDHHC2 had been shown to be crucial PAT associated with SCZ.^28^, ^40^, ^41^ ZDHHC8 and ZDHHC9 were also associated with an increased risk of epilepsy.^42^, ^43^

### 2.2 Functions of protein palmitoylation

**FIGURE 3:**
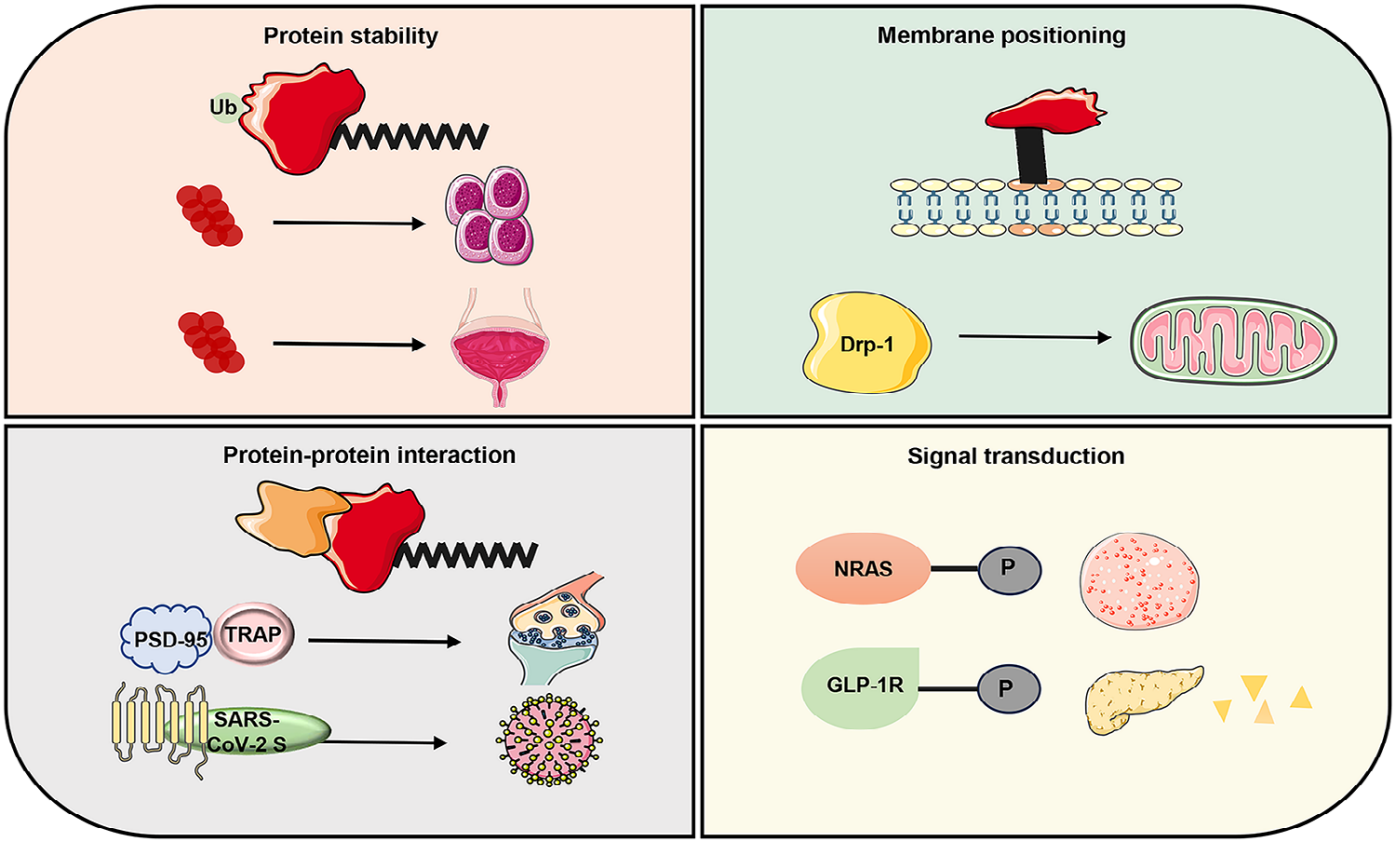
Biological functions of palmitoylation modifications. Palmitoylation modification can improve protein stability, such as protein degradation can accelerate the development of tumor metastasis and bladder cancer; second, palmitoylation modification can change the cell membrane localization, such as the palmitoylation of Drp‐1, which can directly affect the localization and function of the mitochondria; in addition, palmitoylation modification can affect the protein–protein interactions, which can then regulate the synaptic plasticity, and so on; and last, palmitoylation modification can regulate signaling, which is the most important factor in the development of bladder cancer. Targeting other diseases such as leukemia and diabetes. Drp‐1, dynamically related protein 1; PSD‐95, postsynaptic density‐95; SARS‐CoV‐2, severe acute respiratory syndrome coronavirus 2; GLP‐1R, glucagon‐like peptide‐1 receptor; NRAS, neuroblastoma RAS viral onco gene homolog.

Protein palmitoylation, characterized by the attachment of C16 fatty acid chains to Cys residues through a reversible thioester bond, is one of the most prevalent lipid modifications and crucial for regulating protein stability, membrane localization, protein–protein interactions, and diverse cellular signaling pathways (Figure 3).

#### 2.2.1 Protein stability

Increasing evidence indicated that protein palmitoylation contributed to protein stability.^44^ ZDHHC8 intercalates and palmitoylates with SLC7A11 to stabilize it.^45^ Specifically, palmitoylation of Progressive Rod‐Cone degeneration（PRCD）, facilitated by ZDHHC3, was vital for its translocation to the photoreceptor outer segment, with improper localization potentially resulting in retinitis pigmentosa‐related phenotypes.^46^ Furthermore, disruption of flotillin‐1 palmitoylation, whether through mutagenic alterations or competitive peptides, facilitated its degradation, thereby underscoring the critical role of flotillin‐1 palmitoylation in tumor promotion and metastasis induction.^47^ Additionally, ZDHHC9 was found to bind and palmitoylate Bip proteins at the Cys420 site, resulting in enhanced stability of Bip proteins and contributing to therapeutic strategies for bladder cancer.^24^ In addition, cycling of DHHC5 between the dendritic plasma membrane and endosomes contributed to the stability of AMPA channels in the postsynaptic membrane.^25^ ZDHHC9–golgin subfamily A member 7 (GOLGA7) might increase the stability of DHHC9 by preventing its aggregation through the formation of protein complexes.^48^, ^49^ ZDHHC2‐mediated palmitoylation of glycoprotein M6A (GPM6A) was crucial for the regulation of Procr stability, thereby playing an essential role in sustaining stem cell activity.^50^

#### 2.2.2 Membrane positioning

Fatty acids linked by S‐acylation groups usually led to increased thermodynamic and kinetic binding stability of membranes.^51^ APT1 and APT2 S‐palmitoylation were associated with cytoplasmic sol‐membrane transport of their substrates, which in turn promoted altered membrane localization.^52^, ^53^ In addition, S‐palmitoylation is specifically localized at Transmembrane protein 55B（TMEM55B） and TMEM55B‐dependent lysosomes,^54^ and synaptic differentiation induced by the active blockade of gene I(SynDIG1) increased synaptic localization of SynDIG1.^55^ Increased opening of target channels following a conformational change in S‐palmitoylation by improving the interaction of Epithelial Sodium Channels（ENaC） cytoplasmic structural domains with the plasma membrane, while enhancing the membrane affinity of certain channel regions involved in palmitoylation.^56^, ^57^ Moreover, the targeted palmitoylation of glucose transport protein 1 (GLUT1) was crucial for maintaining its localization at the plasma membrane, thereby enhancing glycolytic activity and potentially accelerating the progression of glioblastoma.^58^ DHHC13 was responsible for palmitoylation of dynamically related protein 1 (Drp‐1), which significantly influenced mitochondrial localization and functionality.^59^ Additionally, the palmitoylation of proteins within the claudin family, such as claudin‐5, modulated blood–brain permeability and triggered site‐specific responses to regulate membrane localization.^60^ The small molecule inhibitor of protein palmitoylation, 2‐bromopalmitate (2BP), mitigated hypopharyngeal squamous cell carcinoma by inhibiting the membrane localization of the Ras protein.^61^

#### 2.2.3 Protein–protein interactions

Protein palmitoylation increased the hydrophobicity of protein structural domains, thereby promoting their membrane binding and stabilization, and contributed to protein–protein interactions and mediated subcellular transport between membrane organelles and within specific membrane non‐structural domains.^62^ For example, calcium‐associated proteins could interact with ribosome–transposon complexes and the actin cytoskeleton in S‐acylation to stabilize super‐complexes.^63^ Additionally, ZDHHC could also interact with Severe acute respiratory syndrome coronavirus 2 (SARS‐CoV‐2) spike protein.^64^ Furthermore, the palmitoylation of PSD‐95 was essential for the regulation of synaptic plasticity of α‐amino‐3‐hydroxy‐5‐methyl‐4‐isoxazolepropionic acid receptor (AMPAR).^65^

Sodium‐calcium exchanger 1 (NCX1) exhibits dual functionality; palmitoylation regulated protein–protein interactions by facilitating NCX1 dimerization, while simultaneously influenced the channel affinity of the non‐structured domains of lipid raft (LR) membranes.^66^ In the nervous system, glutamate (GLUT) receptor proteins 1b and 2b (GRIP1b/2b) were palmitoylated by zDHHC5/8, facilitated by the presence of multiple PDZ domains that mediate heterophilic protein–protein interactions.^67^, ^68^ Furthermore, the S‐acylation of CD8 served as a regulatory mechanism, modulating the functional impact of T‐cell activation by fine‐tuning TCR activation thresholds through protein–protein interactions.^69^

#### 2.2.4 Signal conduction

Normal function of numerous surface receptors and signaling proteins required palmitoylation‐mediated for distribution into LRs. In leukocytes, signaling conduction was regulated by palmitoylation.^70^ Specifically, ZDHHC9‐mediated S‐palmitoylation of oncogenic neuroblastoma RAS viral onco gene homolog (NRAS) was indispensable for their localization at the plasma membrane,E and the subsequent activation of downstream signaling pathways that promoted leukemia progression.^71^ Additionally, sodium hydrogen exchanger isoform 1 (NHE1) and other signaling protein‐targeted palmitoylation regulate NHE1 function, which may significantly affect a variety of key cellular functions.^72^ The association of NRAS with the plasma membrane was mediated by palmitoylated at the Cys181 site, and removal of this palmitoylation led to the inactivation of multiple signaling pathways downstream of oncogenic NRAS, thereby suppressing leukemia.^73^ Furthermore, lipid‐induced S‐palmitoylation was an important regulator of cellular signaling.^74^ Palmitoylation influenced the subcellular distribution of BTK isoforms in epithelial tumor cells.^75^ In addition, the palmitoylation of glucagon‐like peptide‐1 receptor (GLP‐1R) at the Cys438 site in response to agonists induced GLP‐1R aggregation, enhanced GLP‐1R signaling, and increased insulin secretion.^76^

## 3 PALMITOYLATION AND NEUROLOGICAL DISORDERS

Recently, there had been a resurgence of interest in targeting protein palmitoylation for the treatment of neurological diseases. Protein palmitoylation had been shown to be involved in therapeutic strategies or mechanisms for AD, PD, HD, SCZ,  Epilepsy, ID, MDD, and NCL. Here, we will review the emerging evidence on the role of protein palmitoylation in various neurological diseases, which provide novel avenues for the treatment of neurological disorders (Table 1).

**TABLE 1:** Palmitoylation targets, sites, and related neurological disorders.

Disease	Pathological features	Targets	Sites	References
AD	Aβ aggregation	APP; BACE1; γ‐secretase; GPM6A; PSD‐95	Cys186, Cys187, Cys474, Cys478, Cys482, Cys485; Cys3, Cys5	^77^ ^77^ ^78^
PD	α‐Syn aggregation; decrease of DA level	SYT11; MAP6; DNAJC5; ERα; DAT	Cys39, Cys40; Cys580	^79^ ^22^ ^80^ ^17^ ^81^
HD	Amplification of CAG trinucleotide repeat sequence in exon 1 of the *HTT* on chromosome 4	HTT; GLT‐1	Cys214	^82^
SCZ epilepsy ID MDD NCL	Reduced thickness of frontal cortex; white matter volume reduction; abnormal discharge of cortical neurons; different genetic and environmental factors pathogenesis is complex abnormal deposition of lipofuscin in nerve cells	GPM6A; ZDHHC5; AMPAR; ZDHHC8; ZDHHC9; ZDHHC9; ZDHHC15; ZDHHC21; ZDHHC7; GLU; ZDHHC5; ZDHHC23	E648; Cys811	^83^ ^84^ ^85^ ^86^ ^87^ ^88^ ^89^ ^90^ ^25^

### 3.1 Palmitoylation and AD

AD is the world's most common neurodegenerative disease, characterized by progressive neuronal degeneration or loss due to excessive accumulation of Aβ peptides, formation of neurofibrillary tangles (NFTs) and hyperphosphorylated tau.^91^ According to epidemiological statistics, 50 million people worldwide are currently living with AD.^92^ An increasing number of studies are now reporting that targeting palmitoylation can modulate AD development.

#### 3.1.1 APP

AD is a progressive neurodegenerative disorder primarily identified by original memory impairment and cognitive decline, which eventually affects behavior, speech, visuospatial orientation, and the motor system.^93^ Its principal pathological features include the accumulation of senile plaques formed by extracellular Aβ deposition and the development of NFTs resulting from hyperphosphorylation of intracellular Tau protein.^94^ Aβ is generated through a series of enzymatic pathways from APP, a type I single‐channel transmembrane protein that undergoes processing and sorting within the ER and trans‐Golgi network.^95^ Upon cleavage by α‐secretase, the neuroprotective soluble APPα (sAPPα) fragment is generated when APP was secreted into the cytoplasmic membrane.^96^ Alternatively, if APP is located within endosome, it can be cleaved by BACE1 to form the sAPPβ fragment.^97^ Subsequently, the transmembrane carboxyterminal fragment could be cleaved by γ‐secretase which results in the formation of neurotoxic Aβ40/42 and APP cytoplasmic protein Aβ42.^98^ Additionally, γ‐secretase cleaves Aβ40/42 and APP intracellular structural domains.^99^

Studies have indicated that approximately 8–10% of APP undergoes palmitoylation,^100^ with Cys186 and Cys187 identified as palmitoylation sites in APP mutants.^77^ Palmitoylation‐deficient APP mutants are sequestered in the ER.^101^ Furthermore, the accumulation of palmitoylated APP (pal APP) in the endogenous mouse brain increased in an age‐dependent manner.^77^ Pal APP is notably localized in LRs, where its palmitoylation promotes the cleavage of APP by BACE1, leading to heightened production of Aβ.^77^ These findings suggest that the specific palmitoylation inhibitors impeded pal APP formation could serve as a promising strategy for the prevention or treatment of AD.^102^ In familial FAD, exome sequencing of affected individuals has identified a new mutation, T209S, located in exon 6 of the *ZDHHC21* gene, and growing studies indicated that the mutated ZDHHC21 T209S protein led to an increase in APP levels.^103^ Specifically, pal APP within LRs is more susceptible to cleavage by BACE1 compared with total APP. It has been observed that 90% of pal APP exists in a dimerized state, whereas only approximately 20% of palmitoylated protein contributes to APP dimer formation.^100^ Protein palmitoylation favors β‐cleavage and facilitates protein dimerization.^104^, ^105^ Moreover, pal APP dimers exhibit a predominance of cis‐formers and demonstrate a 4.5‐fold higher efficiency in dimer formation compared with total APP.^100^ In particular, the formation of sAPPβ–sAPPβ dimers is dependent on APP palmitoylation, whereas the formation of sAPPβ is not. The pal APP dimer may represent a promising target for APP c‐shear inhibitors, given the heightened selectivity of BACE1 for pal APP dimers comparted with total APP dimers.^77^

#### 3.1.2 BACE1

BACE1, a transmembrane aspartic protease necessary for initiating the production of Aβ, has been found to determine axonal transport in neurons of individuals with AD, leading to accumulations in dystrophic neuronal synapses adjacent to cerebral amyloid deposits.^106^ Interaction of proteins with BACE1 may contribute to the development of AD through direct or indirect binding, which can regulate BACE1 at multiple levels, including transcription,^107^ translation,^108^ modification,^109^ and intracellular transport,^110^ thereby influencing the pathogenesis of AD. BACE1 is known to undergo S‐palmitoylation on 4 Cys residues near the membrane. For example, BACE1 has been verified to experience palmitoylation at specific sites within its C‐terminal cytoplasmic and transmembrane domain regions (TMD), including Cys478, Cys482, Cys485, and Cys474 site.^111^ Several enzymes, such as DHHC3/GODZ, DHHC4, DHHC7, DHHC15, and DHHC20, have been identified as PAT responsible for the palmitoylation of BACE1.^39^

At present, the role of BACE1 palmitoylation in Aβ production is contradictory. It has been reported that the absence of the TMD and the non‐palmitoylated form of BACE1 in the cytoplasmic tail can enhance amyloid processing of APP, leading to the generation of Aβ variants distinct from those produced by wild‐type BACE1.^39^ Conversely, when BACE1 is fully localized to LRs through the addition of a glycosyl‐phosphatidyl inositol anchoring site to the exo‐structural domain of BACE1, both sAPPβ secretion and Aβ production were increased.^112^ Overall, the palmitoylated BACE1 was essential for Aβ generation. Another evidence indicated that BACE1 lacking S‐palmitoylation leads to reduced synaptic activity‐dependent Aβ release and decreased brain amyloid burden in vivo.^113^ In addition, whether differences in amyloid load deficits deficient in S‐palmitoylation of BACE1 attenuated memory deficits in 5XFAD mice was investigated.^113^ As expected, the decreased amyloid accumulation resulting from the absence of S‐palmitoylation of BACE1 mitigated cognitive impairments in spatial working memory and associative learning.^113^, ^114^, ^115^ Collectively, these results suggest that the deficiency in S‐palmitoylation plays a vital role in reducing Aβ levels by regulating BACE1 cleavage of APP in endosomes.

#### 3.1.3 γ‐Secretase

Palmitoylation has been shown to impact the activity of two enzymes involved APP metabolism, specifically β and γ‐secretase. Defective S‐palmitoylation of β‐secretase results in reduced Aβ and cognitive deficits,^100^, ^113^ while γ‐secretase cleaves CTFβ (CTF99) to generate amyloid intracellular domain and Aβ following BACE1 processing.^116^ γ‐Secretase is a complicated protein consisting of multiple catalytic subunits, including presenilins, presenilins enhancer 2 (PEN2), anterior pharyngeal defect 1 (APH1), and nicastrin.^117^ S‐palmitoylation of the γ‐secretase subunits, APH1 and nicastrin, has been discerned to regulate PTMs of APP in the brain,^118^ whereas the absence of S‐palmitoylation of APH1 and nicastrin leads to a decrease in Aβ deposition in the brain.^119^ Transgenic mice expressing palmitoylation‐deficient APH1aL and nicastrin showed a marked but moderate reduction in forebrain amyloid deposition and insoluble Aβ levels, suggesting a potential role for γ‐secretase palmitoylation in the regulation of brain Aβ deposition (Figure 3).

#### 3.1.4 GPM6A

GPM6A, a member of the proteolipid protein family, is widely expressed on the surface of neurons in the Central nervous system （CNS）.^120^ Moreover, it plays a crucial role as a signaling molecule in neuronal development, particularly within the hippocampus and cerebral cortex. Also, GPM6A is one of the majors palmitoylated proteins in the brain.^121^ In AD patients, the downregulation of GPM6A expression was observed in hippocampal tissues from AD patients, suggesting that it might be associated with altered synaptic function in the hippocampus of affected individuals.^122^

Extracellular vesicles, including exosomes, macrovesicle, and apoptotic vesicles, are known to contain a variety of nucleic acids, lipids, and proteins.^123^ They can be transferred between cells and are present in bodily fluids, such as blood, urine, and cerebrospinal fluid.^124^ Studies indicated that extracellular vesicles detected in the brain contained pathogenic proteins associated with AD, such as Aβ and tau protein.^125^, ^126^ Quantitative proteomic analyses of extracellular vesicle samples derived from the brains of individuals with AD and healthy control revealed the elevated levels of GPM6A in extracellular vesicles from AD patients.^127^ These findings suggest that the absence of palmitoylated GPM6A reduced the risk of developing AD, and that GPM6A could serve as a potential biomarker to monitor the progression of AD.^127^, ^128^ In addition, studies had demonstrated that selenoprotein K was involved in CD36 palmitoylation via DHHC6, leading to massive phagocytosis of Aβ by microglia, which in turn attenuates the progression of AD, providing novel insights into the therapeutic potential of palmitoyl‐targeted selenoproteins.^15^ Additionally, recent findings indicated that transient receptor potential vanilloid 2 (TRPV2) was palmitoylated at the Cys277 site, and that inhibition of palmitoylation at this site also improved Aβ phagocytosis by microglia.^129^

#### 3.1.5 PSD‐95

PSD‐95 is a scaffold protein derived from postsynaptic dense region, which is encoded by *DLG4* gene and mainly exists in mature and excited GLUT synapses.^130^, ^131^, ^132^ It consisted of three distinct domains, including PSD‐95/Discs large/band occlude protein‐1, Src‐homologue‐3, and guanosine kinase.^133^ Moreover, PSD‐95 can interact with various receptors or signal molecules through these domains, such as N‐methyl‐d‐aspartic acid receptor, potassium channel protein, tyrosine kinase, and cell adhesion molecule, to form a signal complex,^134^ which plays an important role in maintaining synaptic plasticity and regulating learning and memory. PSD‐95 represents the predominant palmitoylated protein in neurons and palmitoylation of PSD‐95 at Cys3 and Cys5 within its N‐terminal domain is crucial for its postsynaptic localization.^78^ PSD‐95 interacts directly with Aβ to modulate synaptic pathology. It has been demonstrated that level of the palmitoylated PSD‐95 was diminished in pathological brain regions of individuals with AD.^135^

**FIGURE 4:**
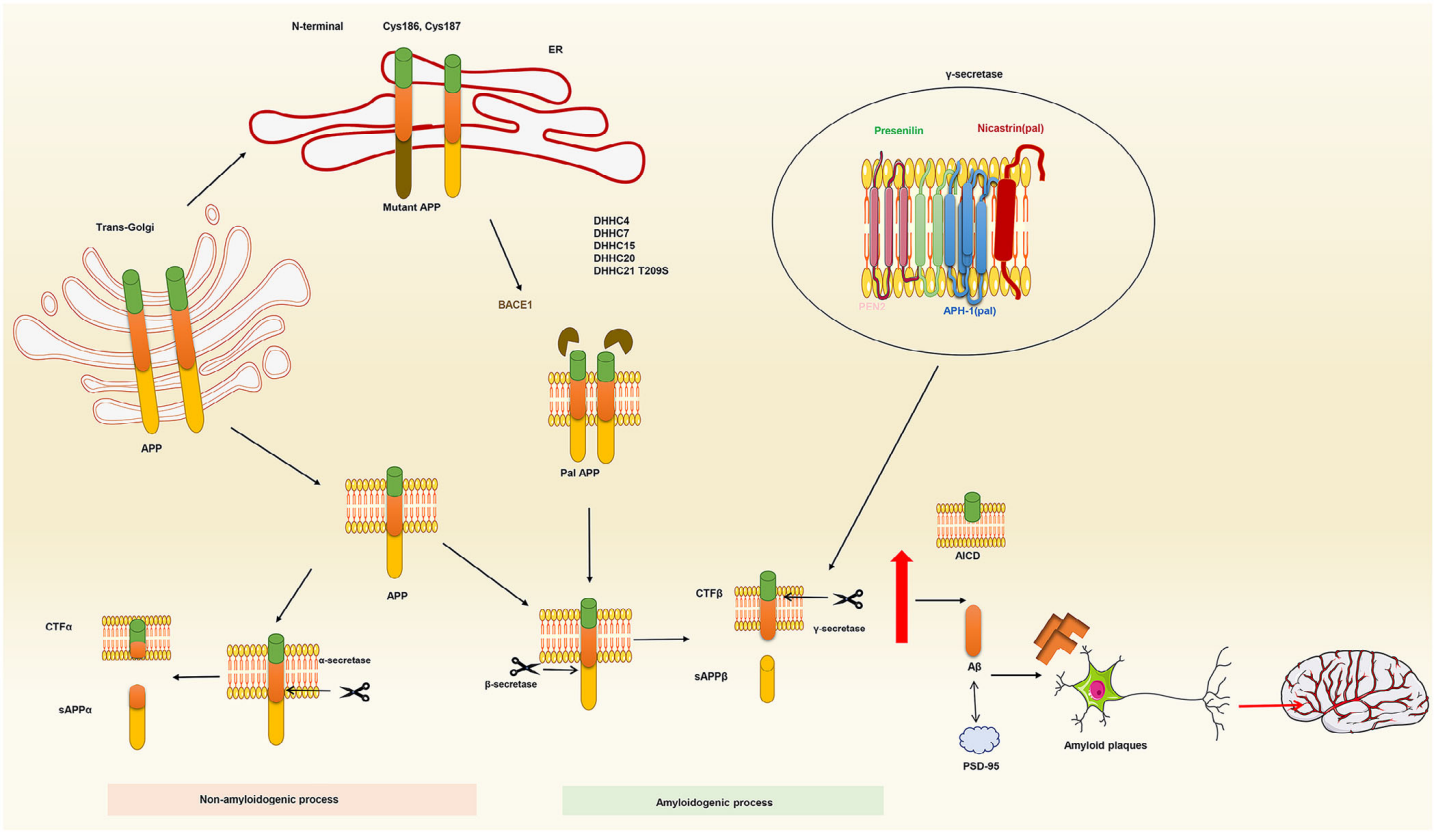
Role of palmitoylation‐related proteins in the pathogenesis of AD. APP translocates from the trans‐Golgi to the plasma membrane and affects the nonamyloidogenic processing of APP in a palmitoylation‐dependent manner. During amyloidosis, APP is palmitoylated at N‐terminal Cys186 and Cys187, and palmitoylation of APP enhances the amyloidogenic pathway that generates Aβ output. APP mutants are then retained in the endoplasmic ER, whereas palmitoylated APP is enriched in the lipid lamellae, leading to upregulation of APP cleavage by BACE1. DHHC4, DHHC7, DHHC15, and DHHC20 promote palmitoylation of BACE1, which in turn promotes APP amyloid processing. Mutated ZDHHC21 T209S protein elevates APP palmitoylation levels, and S‐palmitoylation of two subunits in γ‐secretase, APH1, and nicastrin, increases Aβ deposition in the brain, and additionally, upregulation of PSD‐95 palmitoylation promotes the accumulation of Aβ. Aβ undergoes misfolding to form Aβ plaques that damage neurons. APP: amyloid precursor protein; BACE1, β‐site APP shear enzyme 1; APH1, anterior pharyngeal defect 1; PSD‐95, postsynaptic density‐95; Aβ, amyloid beta; ER, endoplasmic reticulum.

Numerous investigations have indicated that PSD‐95 is a key target of posttranslational palmitoylation‐related signaling pathways, affecting the function of PSD‐95 and its synaptic stability in AD neuropathology.^55^ In addition, PSD‐95 is commonly subject to palmitoylation and elevated level of its palmitoylation has been linked to Aβ accumulation in AD. Furtherly, PSD‐95 palmitoylation was proved to promote the accumulation of Aβ42,^136^ and a decrease in PSD‐95 palmitoylation along with reduced Aβ levels may offer protective effects on synaptic integrity in mice (Figure 4).

### 3.2 Palmitoylation and PD

PD, a prevalent neurodegenerative disorder, is characterized by the progressive loss of dopamine (DA) neurons, usually accompanied by the accumulation and aggregations of α‐syn.^137^ α‐Syn is predominantly expressed in the brain. It is found in the neocortex, hippocampus, substantia nigra (SN), thalamus, and cerebellum.^138^ α‐Syn contains three distinct structural domains, namely, the N‐terminal lipid‐binding α‐helix, the nonamyloid component (NAC), and the C‐terminal acidic tail.^139^ The binding to the C‐terminal region of the DA transporter (DAT) via its NAC sequence has been reported that in α‐syn. This interaction increased the levels of DA in the neurons by promoting the membrane aggregation of DAT and increasing its activity.^140^

#### 3.2.1 Synaptophysin‐11

Synaptophysin‐11 (SYT11), a member of the synaptophysin family, has been identified as a potential risk gene for PD.^141^ It plays a crucial role in regulating vesicle recycling by restricting the location of membrane invagination during early endocytosis. Also, SYT11 inhibits cytokine secretion and phagocytosis to prevent microglia activation under physiological and pathological conditions.^142^, ^143^ Functionally, forebrain‐specific knockout of SYT11 in mice exhibited the impaired synaptic plasticity and memory.^144^ Additionally, increasing studies demonstrated that SYT11 inhibited bulk endocytosis mediated by lattice proteins^143^ and suppressed spontaneous neurotransmission.^145^ Interestingly, SYT11 overexpression in the mouse SN led to defective DA release and the demise of DA neurons.^145^, ^146^ Thus, it was speculated that SYT11 palmitoylation might promote pathological α‐syn aggregation, which in turn aggravated the progression of PD. Furtherly, SYT11 was palmitoylated at positions of Cys39 and Cys40, which were regulated by the lysosome. Moreover, Cys39 and Cys40 of SYT11 played a crucial role in maintaining its stability.^79^ However, SYT11 palmitoylation did not change its subcellular distribution. Collectively, SYT11 palmitoylation could affect α‐syn biology in multiple pathways.

#### 3.2.2 Microtubule‐associated protein 6

Stability of microtubule‐associated proteins is tightly linked to neurons.^147^ Microtubule‐associated protein 6 (MAP6), previously referred to as the STOP protein, is a microtubule‐associated protein that is localized in neurons, astrocytes, oligodendrocytes, fibroblasts, and various other tissues.^148^, ^149^, ^150^, ^151^ Studies had demonstrated a significant correlation between MAP6 and the pathogenesis of PD. Specifically, the glycosylation of MAP6 with Gal‐(β‐1,3)‐GalNAc oligosaccharides facilitates the formation of insoluble inclusion bodies, which subsequently lead to neuronal damage and the disruption of dopaminergic neurotransmission.^152^ Furthermore, the interaction of MAP6 as crucial for the establishment and maintenance of neuronal morphology. During neuronal development, the palmitoylated form of MAP6 was localized in the Golgi apparatus, mitochondria, and plasma membrane^148^, ^153^ as a result of reversible palmitoylation at its N‐terminus,^154^ which facilitated neuronal polarization.^155^

α‐Syn itself could not undergo palmitoylation due to the absence of Cys residues, but it associated with membranes where palmitoylation occurs through an amphipathic helix. In addition, study had reported that the microtubule‐binding protein MAP6 was palmitoylated and translocated to axons via vesicles.^156^ Palmitoylation of MAP6 could transport it to specific subcellular regions by associating with secretory vesicles, and enhance intracellular vesicular transport through its microtubule‐stabilizing function. Conversely, α‐syn disrupted vesicular transport, and α‐syn‐dependent upregulation of MAP6 led to reduced α‐syn inclusion bodies, aberrant α‐syn phosphorylation, and neurotoxicity.^22^ This study provides novel evidence that proteins can exacerbate cellular pathology by facilitating vesicular transport.

#### 3.2.3 DnaJ homolog subfamily C member 5

DnaJ homolog subfamily C member 5 (DNAJC5), also known as Cys string protein α, was a cochaperone of HSC70 and had been demonstrated to regulate the extracellular release of numerous proteins associated with neurodegenerative diseases.^157^ The transgenic expression of α‐syn could protect against progressive neurological disorders upon DNAJC5 depletion.^158^ However, the palmitoylation of DNAJC5 was required for α‐syn secretion. In addition, DNAJC5 stimulated the extracellular secretion of α‐syn by anchoring the protein to the membrane in a DNAJC5 palmitoylation dependent manner.^80^ Consequently, the downregulation of DNAJC5 mutations that are deficient in palmitoylation, resulting in reduced α‐syn accumulation, could serve as an effective strategy for targeting PD.

#### 3.2.4 Estrogen receptor alpha

Estrogen receptor alpha (ERα) is an estrogen‐activated transcription factor involved in regulating gene expression in various organs, including the brain.^159^ Numerous studies had demonstrated that ERα was strongly associated with a variety of neurological disorders.^160^, ^161^ Estrogen had a protective role in PD, as evidenced by case‐control studies indicating that estrogen replacement therapy alleviated PD symptoms in women.^162^ ERα and ERβ undergone palmitoylationare, enabling them to function as membrane signaling proteins in neurons.^163^ Estradiol had been shown to ameliorate synaptic abnormalities mediated by ERα. In vitro studies had demonstrated that reduced ERα distribution is associated with decreased palmitoylation. Since reduced ERα distribution in vitro was accompanied by reduced palmitoylation, a study using a humanized mouse model of 3K α‐syn, oral administration of ML348, a small molecule inhibitor of APT1, over a period of 90 days, demonstrated that palmitoylation positively influenced locomotor and cognitive phenotypes, maintained soluble αS homeostasis, and enhanced synaptic functioning in symptomatic male and female 3K mice, which carry the familial E46K α‐syn mutation linked to PD.^17^ These findings suggested that α‐syn homeostasis and synaptic dysfunction in 3KL mice might have significant functional and pathogenic significance for clinical PD.

#### 3.2.5 DA transporter protein

DA modulation was regulated by the DAT, an integral membrane protein responsible for the reuptake of neurotransmitters from the extracellular space, thereby regulating signaling at both pre‐ and postsynaptic DA receptors.^81^ DAT was predominantly localized to DA neurons that constituted the limbic, mesocortical, and mesostriatal pathways in the midbrain, laying a crucial role in dopaminergic neurotransmission.^164^ Rodent and human DAT contain five conserved Cys residues: Cys6, Cys135, Cys341/342, Cys522/523, and Cys580/581, which are predicted to be exposed to the cytoplasm, and therefore represent potential S‐palmitoylation sites. Notably, palmitoylation at Cys580 contributed to the enhanced kinetic capacity of these transporter proteins. Furthermore, the overexpression of DHHC enzymes in the rDAT cell system led to a dramatic increase in the transporter's V_max, driven by a kinetic mechanism. Conversely, prolonged inhibition of palmitoylation led to the degradation of DAT.^81^ Palmitoylation is crucial for the regulation of DAT function and presents a potential therapeutic target for maintaining DA homeostasis in dopaminergic disorders. Furthermore, the palmitoyl group found in the cortex of PD patients might play an important role in the pathophysiology of PD.^165^ Investigating the role of palmitoyl‐proteomics in PD could provide insights into novel therapeutic targets.

### 3.3 Palmitoylation and HD

HD is an inherited neurodegenerative disorder commonly characterized by neuropsychiatric symptoms, chorea‐like movement disorders and progressive cognitive deficits.^166^ HD is caused by an amplified polyglutamine‐encoding (CAG) trinucleotide repeat in the HTT gene,^167^ and there is no cure or palliative care for the disease. Research suggests that HD may be in part a disease of altered palmitoylation.^168^

#### 3.3.1 HTT

HD is an autosomal dominant disorder characterized by an expansion of the CAG trinucleotide repeat sequence in exon 1 of the *HTT* gene on chromosome 4.^169^ As a risk factor of HD, HTT is typically modified at Cys 214, a modification crucial for its intracellular transport.^82^ Huntingtin‐interacting protein 14 (HIP14) is a mammalian ortholog of Akr1p that is indispensable for protein palmitoylation. HIP14, involved in the regulation of protein trafficking, has been identified as an interacting protein of HTT.^170^ Numerous studies have shown that HIP14 plays an important role in regulating HTT palmitoylation.^16^ For example, overexpression of HIP14 caused endogenous HTT and, to a lesser extent, mutant HTT to relocalization to the Golgi apparatus. This relocalization did not take place in HTT that was resistant to palmitoylation, indicating that trafficking of HTT to the Golgi was influenced by palmitoylation.^171^ Furthermore, HIP14 overexpression led to an obvious increase in the palmitoylation of both wild‐type and mutant HTT, suggesting that HIP14 was involved in the catalysis of HTT palmitoylation.^172^

In addition, HIP14 can modulate the distribution of specific subsets of palmitoylated proteins in a palmitoylation‐dependent manner.^171^ For instance, HIP14 overexpression resulted in an apparent reduction in the presence of HTT inclusions in polyglutamine (polyQ) expansion, but not in palmitoylation‐resistant mutants, suggesting that HIP14‐medicated HTT palmitoylation was critical for intracellular distribution and trafficking.^16^ Notably, the absence of HTT palmitoylation exacerbated toxicity, leading to the widespread protein aggregation and cell death.^171^

**FIGURE 5:**
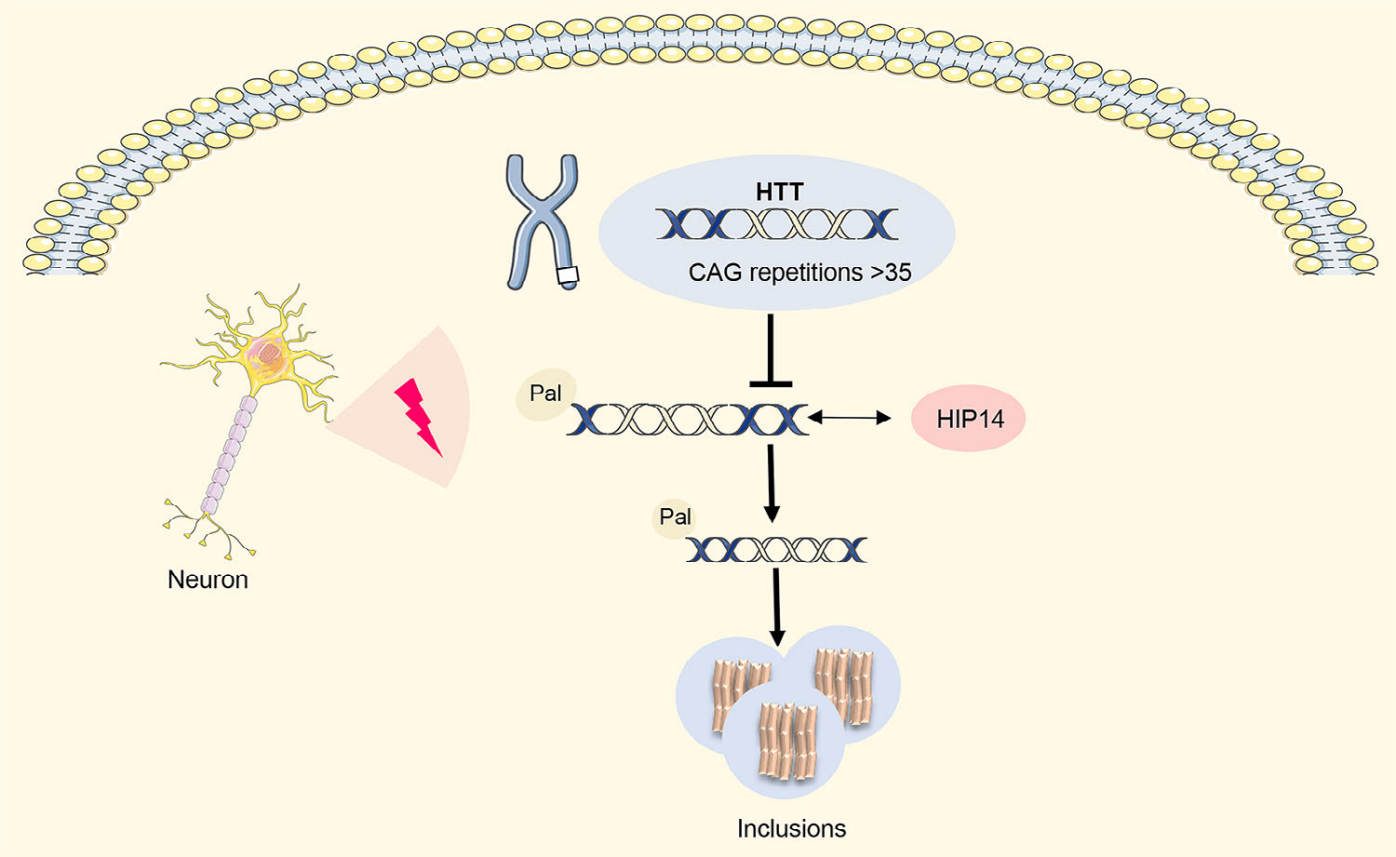
HIP14 plays an important role in regulating HTT palmitoylation. CAG triplet repeat mutations (>35 repeats) in *HTT* gene on chromosome 4 inhibits the interaction of mutant HTT and HIP14, leading to a significant reduction in HTT palmitoylation. Downregulation of palmitoylated HTT accelerates the formation of inclusions and damages neuronal cells. HIP14, Huntingtin‐interacting protein 14; HTT, Huntingtin.

Besides, mutations in the palmitoylation site of HTT caused resistance to palmitoylation, thereby hastening the formation of inclusions and increasing neuro toxicity. Downregulation of HIP14 in neurons of mice expressing both wild‐type and mutant HTT led to an increase in the formation of mutant HTT inclusions, whereas overexpression of HIP14 reduced the presence of inclusions (Figure 5). Expansion of swollen polyQ bundles in HTT decreased palmitoylation, further contributing to the formation of inclusions and exacerbating neuro toxicity. Another evidence indicated that HTT proteins in the brains of individuals of HD exhibited the markedly diminished levels of palmitoylation as a result of variations in the modification sites.^173^ This reduction in palmitoylation promoted the overexpression of polyQ, facilitating the rapid formation of inclusion bodies and increased neurotoxicity.^16^ The APT1 inhibitor ML348 increases BDNF transport and brain palmitoylation, which in turn improves behavior and pathology in HD mice.^174^

#### 3.3.2 Glutamate transporter‐1

Glutamate transporter‐1 (GLT‐1), also known as excitatory amino acid transporter 2, is a crucial factor in regulating extracellular GLUT concentrations to prevent neurotoxicity.^121^ Dysregulation of GLT‐1 has been confirmed to be closely associated with excitotoxicity, neuronal death, and neurological disorders.^175^ For example, the reduced mRNA levels of GLT‐1 and impaired GLUT uptake were observed in HD postmortem and R6/2 mice.^176^ In addition, increasing GLT‐1 expression could mitigate behavioral abnormalities in the R6/2 transgenic model. Moreover, the decreased GLT‐1 palmitoylation was discerned in HD mouse model.^177^ Elsewise, STHdh‐Q111 cells expressed full‐length mutant HTT with 111 CAG repeats also caused an obviously reduction of GLT‐1 palmitoylation.^177^ Then, the impaired GLT‐1 palmitoylation could contribute to the decreased GLUT uptake, excitotoxicity and, ultimately, neuronal death in HD.

### 3.4 Palmitoylation and SCZ

SCZ is a disabling illness that affects approximately 1% of the world's population.^178^ The pathogenesis of SCZ is characterized by neurotransmitter dysregulation, immune disturbances, and abnormal neural development.^179^ Antipsychotics are the first‐line treatment for SCZ, but have serious side effects. There is currently no curative treatment available. However, palmitoylation may be closely linked to the pathogenesis of SCZ.

#### 3.4.1 GPM6A

SCZ is a severe neuropsychiatric disorder with aberrant social behavior and widespread cognitive deficits. It is thought to result from abnormal brain development in the first 30 years of life,^180^ affecting approximately 1% of the global population.^181^ Genome‐wide association studies (GWAS) have identified GPM6A as a potential causative gene for SCZ, particularly for the depressive subtype. Moreover, analysis of genetic data from 280 patients with SCZ and 525 healthy controls has revealed a close association between GPM6A and the depressive subtype of SCZ.^182^ Meanwhile, GPM6A has been linked to stress response and alterations in hippocampal volume,^183^, ^184^ which are commonly observed in patients with SCZ.^185^ Taken together, these collective findings support that GPM6A might influence SCZ by impacting brain development via GPM6A palmitoylation.

#### 3.4.2 ZDHHC proteins

ZDHHC proteins are related to palmitoylation modification after protein's translation, and most of these proteins have the activity of PAT.^186^ Studies have shown that palmitoylation regulated by ZDHHC protein is closely associated with human diseases.^187^ Recently, the relationship between SCZ and the ZDHHC family of proteins has received much attention. For example, DHHC8‐deficient mice had deficits in SCZ‐related circuits, prefrontal cortex (PFC)–hippocampal connectivity, and spatial working memory.^188^ Furthermore, GWAS have shown that ZDHHC5 is a candidate gene for SCZ.^20^ In particular, a novel mutation in ZDHHC5 was identified in a patient with SCZ, resulting in a premature stop codon at residue 648 (E648) and the loss of the last 68 amino acids of ZDHHC5,^83^ including the PDZ binding motif.^189^, ^190^ Furthermore, it has been shown that ZDHHC5 was required for spine maturation and stability,^190^ suggesting that immature synapses might be eliminated prior to maturation. Together, ZDHHC5 may be implicated in the development of excitatory synapse by integrating its palmitoylation activity, C‐terminal structural domains, and surface localization.^190^ Moreover, ZDHHC2, a member of the ZDHHC protein family, is responsible for palmitoylation of PSD‐95 and a‐kinase‐anchoring protein 79/150 (AKAP79/150).^191^ Analysis of targeted sequencing data from 1827 SCZ patients and 1004 healthy controls identified specific variants of ZDHHC2, including rs73198534, rs530313445, and rs74406481, that were associated with an increased risk of SCZ.^23^

### 3.5 Palmitoylation and epilepsy

Seizures affect the lives of 10% of the world's population^192^ and are a disorder characterized by an underlying susceptibility to seizures and their neurobiological, cognitive, psychological, and social consequences. Medication is currently the preferred option for epilepsy treatment; however, this therapy only controls or relieves symptoms. Study suggests palmitoylation may sharpen pathogenesis of epilepsy.^193^

#### 3.5.1 α‐Amino‐3‐hydroxy‐5‐methyl‐4‐isoxazolepropionic acid receptor

AMPAR, a subtype of the ionotropic GLUT receptors, plays a prominent role in neurotransmission and is widespread throughout the CNS.^194^ AMPAR has long been regarded as a potential pharmacological target for epilepsy treatment.^195^ Studies have confirmed that reversible palmitoylation of the carboxyl terminus is an important mechanism for regulating the postsynaptic translocation of AMPAR.^196^ For example, 2BP palmitoyl‐transferase inhibitor ameliorated the aberrant neural activity resulting from AMPAR palmitoylation, suggesting the potential application of palmitoylation inhibitors in the prevention of epileptic seizures.^197^, ^198^

Structurally, AMPAR is composed of four subunits, namely GluA1, GluA2, GluA3, and GluA4 (also referred to as GluR1‐4, GluRA‐D, or GluRa1‐4).^199^ The subunit composition of AMPAR determines the transport pathway of AMPAR, with GluA1 exhibiting greater dominance compared with the other subunits in the activity‐dependent cytosol to synapse trafficking of AMPAR.^200^ It has been shown that the palmitoylation of the GluA1 subunit in postsynaptic AMPAR could inhibit the synaptic induction of long‐term potentiation (LTP), thereby serving as a protective mechanism against seizures.^193^ Also, aberrant palmitoylation of GluA1 led to hyperexcitation of the brain and disruption of neural transmission, which in turn leads to seizures [114].

In addition, AMPAR palmitoylation may hold potential in reducing resistance to antiepileptic medications.^193^ Studies have demonstrated that epileptic mice developed resistance to antiepileptic drugs, including sodium valproate, phenobarbital, and diazepam. These drugs targeted GABAergic neurotransmission when the palmitoylation site at Cys811 of the GluA1 subunit was substituted with serine.^84^ This suggested that disruptions in AMPAR palmitoylation might impact the effectiveness of antiepileptic drugs. Similarly, mice lacking the C‐terminal palmitoylation site of GluA1 at Cys811 presented that the absence of palmitoylation increased susceptibility to seizure and led to LTP‐induced spinal enlargement, while not affecting overall brain structure or typical excitatory synaptic transmission. These findings implied that aberrant palmitoylation‐dependent regulation of AMPAR could contribute to the enhanced excitability and subsequently disrupt network stability, ultimately culminating in seizures activity.^193^ Besides, the compound N‐tert‐butyl hydroxylamine (NtBuHA), a potential depalmitoylating agent, caused the depalmitoylation of PSD‐95 and AKAP79/150, and then reduced the distribution and synaptic activity of AMPAR at the postsynaptic membrane in the hippocampus.^201^ These findings suggested that NtBuHA might have diverse therapeutic effects not only on epilepsy, but also on other neurological disorders associated with abnormalities in GLUT signaling.^202^

#### 3.5.2 ZDHHC proteins

Previous studies revealed that hemizygous deletion of the *ZDHHC8* gene might be associated with SCZ.^203^ Furtherly, in the brains of temporal lobe epilepsy patients, ZDHHC8 expression was increased, similar to that observed in chronic epileptic mice, suggesting that ZDHHC8 was correlated with human epilepsy.^42^ In addition, the levels of ZDHHC8 in mice with spontaneous recurrent seizures were associated with the susceptibility to seizures.^100^

Another member of the ZDHHC family, ZDHHC9, was verified to be associated with an increased risk of epilepsy.^43^ Nine males from 3 families who possessed loss‐of‐function mutations in ZDHHC9 were diagnosed with epilepsy.^204^ Moreover, the epileptic histories and electroencephalographic characteristics of the ZDHHC9‐mutant individuals exhibited similarities to rolandic epilepsy (RE), indicating a potential association between ZDHHC9 mutations and susceptibility to focal epilepsy, along with distinct cognitive deficits consistent with the RE spectrum.^204^ In addition, ZDHHC9 facilitated dendritic growth and inhibited synapse formation by palmitoylating 2 different substrates, the small GTPases N‐Ras and TC10.^43^ Depletion of ZDHHC9 in rat hippocampal resulted in shorter and less dendritic spindles complexity and an increased ratio of excitatory to inhibitory synapses on mutant cells.^43^ This was supported by evidence from in vivo studies showing that ZDHHC9 knockout mice exhibited spontaneous high‐frequency spiking activity indicative of nonconvulsive seizures.^85^ Meanwhile, in vitro studies using acute brain slices revealed that Cornu ammonis（CA1） hippocampal neurons presented an increased frequency and amplitude of spontaneous excitatory and inhibitory postsynaptic currents, as well as the enhanced small excitatory and inhibitory presynaptic currents.^43^ These results underscored the role of ZDHHC9 in the development of neural circuits and established a connection between loss‐of‐function in ZDHHC9 and the occurrence of epilepsy in patients with X‐linked ID.

### 3.6 Palmitoylation and ID

ID is a neurodevelopmental disorder affecting approximately 1–3% of the global population.^205^ It is a genetically heterogeneous condition, with 1679 human genes currently associated with its phenotype.^206^ Typically manifesting before the age of 18, ID is characterized by significantly lower intelligence than the general population,^207^ which substantially impairs cognitive functioning, daily living skills, and social interaction.^208^ These impairments are predominantly attributed to genetic factors.^209^

Research had identified variations in the ZDHHC9 gene as a causative factor for Raymond type X‐linked ID (XLID).^210^ Loss of function of ZDHHC9 was associated with XLID.^211^ Mutations at the ZDHHC9 Xq26.1 locus, the identified first palmitoyl transferase to target ID, were responsible for X‐Linked mental retardation（XLMR） palmitoylation.^18^ Behavioral deficits observed in ZDHHC9 mutant mouse were consistent with those reported in mouse models with ID gene mutations.^212^, ^213^, ^214^ It was determined that the behavior of these mouse model was influenced by the ZDHHC9 gene associated with mental retardation.^85^ ZDHHC9 R148W undergone auto‐palmitoylation driven by burst kinetics, while the P150S mutation in ZDHHC9 reduced the initial burst period of the auto‐palmitoylation reaction. These two mutations alter palmitoylation activity through distinct mechanisms.^215^ GCP16 could act as an accessory protein to activate DHHC9, GOLGA7, a small palmitoylated peripheral membrane protein that bound to proteins involved in Golgi vesicle trafficking.^216^ In contrast to GCP16, GOLGA7B, had been demonstrated to be a highly effective protein, despite not enhancing the stability of other members of the DHHC9 subfamily.^48^ Research indicated that GCP16, within the DHHC9–GCP16 complex, significantly elevated DHHC9 protein levels and hold the potential to target XLID.^48^

ZDHHC15 was one of several genes on the X chromosome that are duplicated in individuals with intellectual disabilities.^217^ Its expression is disrupted by an X chromosome ectopic in an intellectually disabled woman.^86^ Furthermore, ZDHHC15 knockdown could influence the number of dopaminergic neurons in zebrafish.^87^ ZDHHC15‐specific RT‐PCR analysis of the patient's lymphocytes identified a variant of the ZDHHC15 transcript, and the absence of this transcript contributed to the XLID phenotype.^86^

### 3.7 Palmitoylation and MDD

MDD is a prevalent neuropsychiatric disorder that imposes significant economic and social burdens, with high prevalence.^218^ Potential mechanisms associated with MDD include neurotransmitter imbalances, inflammation, and neural network dysfunction.^219^ Studies had demonstrated that palmitoylation modulated depressive‐like behaviors in mice, suggesting that targeting palmitoylation could represent a novel therapeutic strategy.

#### 3.7.1 ZDHHC proteins

Research indicates that the expression and palmitoylation levels of DHHC2 are elevated following chronic restraint stress (CRS). Knockdown of DHHC2 could prevent CRS‐induced depressive‐like behaviors and attenuate AKAP150 signaling and synaptic transmission in the BLA of mice.^219^ 5‐HT1A, a subtype of serotonin receptor, is typically expressed in the hippocampus, septum, amygdala, and PFC,^220^, ^221^ where it is abundant.^222^ Palmitoylation of 5‐HT1A by ZDHHC21 led to depressive‐like behavior in rodents models. Additionally, 5‐HT1A was reduced in human and rodent brain.^88^ Study indicated that C3aR activation might accelerate the onset of MDD.^223^ C3aR, a receptor for complement C3, could block the excessive activation of the inhibitory microglia APT2/DHHC7 palmitoylation cycle, which mediated STAT3 translocation and the expression of pro‐inflammatory cytokines. Furthermore, the activation of STAT3, facilitated by the C3aR palmitoylation cycle, might serve as a predictive marker for the onset of depression.^89^

#### 3.7.2 GLUT

GLUT levels downregulation could protect against neurotoxicity and neurological damage in depression.^224^ Fluoxetine, a selective serotonin reuptake inhibitor, modulated glucose metabolism by regulating the palmitoylation of glucose transporter proteins. Specifically, fluoxetine significantly enhances the palmitoylation of GLUT3 and robustly induces the palmitoylation of GLUT1 in human peripheral blood monocytes and N2a cells. These findings suggest that enhancing cerebral glucose uptake could represent an effective therapeutic strategy for managing MDD.^90^

### 3.8 Palmitoylation and NCL

NCL is a neurodegenerative disease associated with cognitive decline, progressive cerebellar atrophy, retinopathy, and myoclonic epilepsy,^225^ characterized by the accumulation of spontaneous fluorescent ceroid lipochromes in most cells. Clinical features of NCL include vision loss, seizures, psychomotor degeneration,^226^ and premature death.^227^

Study demonstrates that targeting palmitoylation may accelerate the development of NCL.^228^

Fourteen genes (CLN1‐14) had been identified to be associated with NCL,^229^ a disease attributed to mutations in the gene encoded by PPT1.^230^ It had been proposed that PPT1 mutations might dysregulate Ca++ transport from the ER to the lysosome, thereby disrupting lysosomal Ca++ homeostasis and inhibiting the catalytic activity of Ca++ dependent lysosomal hydrolases, leading to impaired degradation of undigested cargoes in the autophagosome, and consequently accelerating the onset of NCL.^231^ Dysregulation of palmitoylation contributed to NCL development by a comparative analysis of the palmitome in control and DNAJC5/CLN4 patient brains.^19^ Reduced levels of ZDHHC5 and ZDHHC23 could inhibit membrane‐bound APT1, which in turn localizes H‐Ras at the plasma membrane, activating signaling pathways for microglia proliferation and exacerbating neurotoxicity.^25^ Furthermore, the administration of NtBuHA to CLN1 mice, as opposed to PPT1, resulted in improved neuroinflammation. These findings suggest that NtBuHA exerts a neuroprotective effect and may serve as a potential target for therapeutic intervention.^232^ In addition, a novel mutation carrying the DNAJC5 gene p.C128Y led to aberrant palmitoylation and triggered lipofuscin deposition.^157^

## 4 PALMITOYLATION AND OTHER HUMAN DISEASES

In addition to neurological disorders, studies had indicated that protein palmitoylation was also implicated in a wide range of diseases, such as cancers, cardiovascular diseases, metabolic disease, and infectious diseases, among others.^118^, ^233^, ^234^, ^235^ Specifically, TGR5 deletion could promote CD36 localization at the plasma membrane by upregulating of CD36 palmitoylation mediated by DHHC4, leading to increased fatty acid uptake and lipid accumulation in cardiomyocytes.^236^ In myocardial ischemic injury, palmitoylation of gasdermin D (GSDMD) at Cys192 facilitated its cytosolic localization through ZDHHC14, thereby exacerbating focal death in cardiomyocytes.^237^ Disulfiram had been shown to antagonize palmitoylation at this site, suggesting that targeting GSDMD Cys192 palmitoylation with disulfiram could serve as a potential therapeutic strategy for myocardial cell death.^238^ Recently, study had also indicated that deletion of ZDHHC1 significantly diminished the palmitoylation of p110α, promoted its translocation to the nucleus, and limited the PI3K‐Akt‐mTOR signaling pathway. This process attenuated the differentiation of monocytes into macrophages, reduced lipid uptake, and ultimately impeded the progression of atherosclerosis.^239^

Interferon‐induced transmembrane proteins (IFITMs) are recognized as broad‐spectrum antiviral factors that inhibit the entry of various viruses into host cells.^240^ It has been shown that site‐specific S‐palmitoylation of Cys72 was crucial for the trafficking of IFITM3 to restrict viral infection such as influenza A virus,^241^ and Japanese encephalitis virus.^118^ In addition, Severe acute respiratory syndrome (SARS‐CoV‐2) spike protein determined viral entry, and its palmitoylation could affect viral infection.^242^ Knockdown of the ZDHHC9 had been shown to reduce SARS‐CoV‐2 fusions and infections.^243^ Furthermore, palmitoylation of the SARS‐CoV‐2 envelope protein by ZDHHC3, 6, 12, 15, and 20 at Cys40, Cys43, and Cys44 reduced the stability of the envelope protein and the density of virus‐like particles (VLPs), demonstrating that palmitoylated envelope protein was central to the normal morphogenesis of SARS‐CoV‐2 VLPs.^244^ Angiotensin‐converting enzyme 2 (ACE2), previously identified as the cellular receptor for SARS‐CoV, undergone S‐palmitoylation at Cys141 and Cys498 by ZDHHC3, which was crucial for its membrane targeting and extracellular vesicle secretion.^245^

It had been shown that targeted palmitoylation might contribute to the treatment of cancer. For instance, knockdown of ZDHHC12 also significantly inhibited the precise membrane localization of the membrane protein claudin‐3, affecting its stability and reducing tumorigenesis in ovarian cancer cells.^246^ Similarly, palmitoylation of malate dehydrogenase 2, catalyzed by ZDHHC18, was essential for maintaining mitochondrial respiration and promoting malignancy in ovarian cancer.^247^ Additionally, ZDHHC22‐mediated mTOR palmitoylation and claudin‐6‐mediated RAS palmitoylation via SREBP1 inhibited breast cancer growth.^248^, ^249^ In pancreatic cancer, ZDHHC9 played pivotal role by regulating PD‐L1 through palmitoylation, and its inactivation could serve as an effective immunotherapeutic strategy to enhance anti‐PD‐L1 therapy.^250^

Overall, these findings underscore the widespread involvement of palmitoylation in the pathogenesis of human diseases. However, most of the studies are limited to the protein level under physiopathological conditions. Currently, the development of drugs targeting palmitoylation for the treatment of human diseases faces numerous challenges. Future efforts should focus on achieving higher levels of scientific innovation and technological advancement to discover more effective therapeutic strategies.

## 5 CURRENT OCCURRENCE AND CHALLENGES FOR NEUROLOGICAL DISEASE THERAPY

Neurological disorders pose a substantial global health challenge, and constitute the primary cause of disability, ranking second only to cardiovascular diseases.^251^ Moreover, the incidence of neurological disorders has escalated significantly over the past few decades. Currently, therapeutic strategies for neurological disorders include stem cell therapy,^252^ gene therapy,^253^ RNA‐based therapies,^254^ among other. However, these interventional strategies are primarily restricted to alleviating symptoms and postponing the onset and progression of the disease. Neurological disorders represent one of the most formidable therapeutic areas for achieving successful drug approval.^255^ One of the challenging obstacles in the delivery of therapeutics for neurological conditions is the blood–brain barrier (BBB), and novel drugs that can successfully cross the BBB to target neurological diseases pose a substantial challenge for researchers and clinicians. Identifying targeted therapies for clinical application remains difficult, given the incomplete understanding of the precise pathogenesis of many neurological disorders. Gleaning insights from unsuccessful clinical trials and persistently advancing novel therapeutics tailored to clinical demands, with a focus on multimechanism and multitargeting strategies, will remain a pivotal theme in achieving breakthroughs in the treatment of neurological diseases.

## 6 PRECLINICAL EXPERIMENTS AND CLINICAL TRIALS

Preclinical studies using animals to evaluate the potential of therapeutic drugs or strategies are critical precursors to clinical trials. Currently, few preclinical studies focusing on interventional strategies that target protein palmitoylation. Studies had indicated that enzyme replacement therapy was a prevalent therapeutic approach for neurological diseases. For example, repeated administration of recombinant human PPT1 had been shown to be an effective treatment for NCL in both murine and ovine models.^256^ In preclinical animal studies examining PPT1‐associated NCL, the effects of high‐dose intravenous injection of human recombinant PPT1 were investigated with respect to alterations in motor performance, survival rates, and neuropathological changes in the brain. This treatment was well tolerated, as evidenced by the absence of antibody formation and allergic reactions.^257^ Furthermore, the administration of NtBuHA in CLN1 mouse models, as an alternative to PPT1, resulted in an amelioration of neuroinflammation. These findings indicate that NtBuHA may exert neuroprotective effects and holds potential as a therapeutic target in drug development.^258^

Cholesterol acyltransferase (ACAT) inhibitor led to impaired APP processing, while APP palmitoylation is known to enhance amyloidogenic processing. Consequently, targeting ACAT or employing specific palmitoylation inhibitors represents a promising therapeutic strategy for the treatment or prevention of AD.^77^ Furthermore, the palmitoylated prolactin‐releasing peptide (palm 11–PrRP31) reduced Aβ plaques and attenuated neuroinflammation in the APP/PS1 mouse model,^259^ suggesting its potential as an effective therapeutic agent targeting palmitoylation in AD. Toxicity‐reducing MTT therapies have demonstrated potential in preclinical animal studies for HD.^260^, ^261^ Additionally, disrupting the interaction between APT and HTT or HIP14 might represent a promising strategy for advancing HD treatment.^173^

## 7 POTENTIAL INTERVENTIONAL STRATEGY TARGETING ZDHHC PROTEINS

Currently, there is an increasing interest in targeted palmitoylation in the treatment of neurological diseases, although no clinically approved targeted drugs are available. ZDHHCs, the crucial enzymes responsible for catalyzing palmitoylation process, play important regulation roles in neurological disorders. More specially, ZDHHC6 mediated palmitoylation of selenoproteins K was involved in the inhibition of Aβ phagocytosis.^15^ Similarly, inhibition of TRPV2 palmitoylation catalyzed by ZDHHC21 could also improve Aβ phagocytosis.^129^ In addition, aberrant palmitoylation resulting from the ZDHHC21 p.T209S mutation might represent a novel pathogenic mechanism, and its inhibitor could potentially alleviate the progression of AD.^103^ Additionally, CEGI might affect Aβ deposition by inhibiting PSD‐95 palmitoylation.^135^ Deletion of DHHC2, DHHC3, DHHC8, DHHC15, and DHHC17 led to decreased DAT palmitoylation, which in turn reduced DAT stability and disrupted DA transport.^258^ DHHC17 and DHHC13 deletion could also lead to the diminished palmitoylation of HTT, exacerbating the progression of HD.^262^ Mutations in ZDHHC5 at position E648 and ZDHHC2 at rs73198534, rs530313445, and rs74406481, increased the risk of the development of SCZ.^23^, ^83^ Furthermore, downregulation of AKAP150 palmitoylation mediated by ZDHHC2 and upregulation of 5‐HT1A palmitoylation could be an effective strategy for the treatment of MDD.^219^ ZDHHC9 was the first PATs identified to be associated with ID.^18^ Study showed that co‐expression of ZDHHC9 and GCP16 increased ZDHHC9 stability, suggesting its potential role in mitigating the risk of ID.^48^ Additionally, ZDHHC9 palmitoylation could also inhibit synapse formation, and mutations at the ZDHHC9 locus were associated with an increased susceptibility to epilepsy^43^, ^204^ (Table 2).

**TABLE 2:** Potential interventional strategy targeting ZDHHC proteins.

Diseases	ZDHHC isoforms	Pathogenic mechanism	Targeted therapeutic strategies	References
AD	ZDHHC6	selenoproteins could inhibit Aβ phagocytosis through ZDHHC6 participation in	Selenoproteins as potential therapeutic agents	^15^
	ZDHHC6	Blocking TRPV2 palmitoylation improves Aβ phagocytosis	Modulation of TRPV2 channel sensitivity could be a potential therapeutic strategy	^129^
	ZDHHC21	Increased APP palmitoylation leads to Aβ production in ZDHHC21T209S/T209S mice	–	^103^
	ZDHHC21	CEGI may affect Aβ deposition by inhibiting PSD‐95 palmitoylation	Inhibition of PSD‐95 palmitoylation may be a potential therapy for AD treatment	^135^
PD	DHHC2; DHHC3; DHHC8; DHHC15; DHHC17	DHHC2, DHHC3, DHHC8, DHHC15, and DHHC17 stimulate DAT palmitoylation to increase the stability of dopamine transporter protein (DAT)	–	^258^
HD	DHHC17; DHHC13	Deletion of DHHC17 and DHHC13 leads to reduced palmitoylation of HTT aggravating HD development	–	^262^
SCZ	ZDHHC5; ZDHHC2	Site‐specific variation in ZDHHC2 and ZDHHC5	Regulation of ZDHHC2 and ZDHHC5 stability	^23^, ^83^
Epilepsy	ZDHHC9	Site variation in ZDHHC9	Regulation of ZDHHC9 stability	^204^
ID	ZDHHC9	Site variation in ZDHHC9	Regulation of ZDHHC9 stability; targeting the DHHC9–GCP16 complex	^18^, ^48^
MDD	ZDHHC2	DHHC2 knockdown attenuates AKAP150 signaling and synaptic transmission in the BLA of mice	Furthermore, downregulation of ZDHHC2 targeting the AKAP150 palmitoylation signaling pathway	^219^
	ZDHHC21	Decreased palmitoylation of 5‐HT1A	Upgraded 5‐HT1A palmitoylation	^88^
NCL	ZDHHC5; ZDHHC23	Reduced levels of ZDHHC5 and ZDHHC23 inhibited APT1 and enhanced neurotoxicity	NtBuHA instead of PPT1 treatment	^232^

Overall, the advancement of novel interventions aimed to ameliorate neurological disorders via targeting palmitoylation process presents a consistently formidable technical challenge. An in‐depth exploration of the relationship between aberrant palmitoylation and the pathogenesis of neurological disorders could contribute to the development of new therapeutic and preventive strategies for neurological disorders.

## 8 CONCLUSION AND PROSPECTS

Mechanistically, dysregulation of palmitoylation or depalmitoylation might represent a critical factor influencing neurological diseases, and protein acyltransferases PATs and PPT1, especially APT1 and APT2, have been closely linked to neurological disease. In addition, ZDHHC proteins, which facilitate palmitoylation, are also implicated in the pathophysiology of these diseases. Palmitoylation of both presynaptic proteins and postsynaptic receptors regulated synaptic protein transport and localization. Therefore, to address the limitations of current targeted palmitoylation therapies, it is crucial to elucidate the complex mechanisms underlying functional synaptic regulation, explore the potential pathways of palmitoylation and depalmitoylation, and utilize the catalytic mechanism of ZDHHC enzymes as a basis for developing ZDHHC inhibitors.

Given the reversible nature of protein palmitoylation, understanding its regulatory mechanisms represent a significant biological challenge. Recently, the increasing recognition of PTMs has highlighted their potential interdependence and interactive dynamics, which collectively regulate biological functions through antagonistic or synergistic effects. Notably, glycosylation, phosphorylation, S‐nitrosylation, and ubiquitination are all subject to crosstalk regulation with palmitoylation. For example, the bidirectional crosstalk between Syn1 palmitoylation and phosphorylation modulates synaptic vesicle kinetics in neurons, while the interplay between protein palmitoylation and nitrosylation orchestrates anxiety‐related behaviors in rats. Similarly, crosstalk between palmitoylation and specific protein modifications might be a present a potential target for the treatment of neurological disorders. These interventional strategies involve the identification of intercalating proteins, the confirmation of corresponding modifications on the target protein by the intercalating protein, and ultimately the validation of the associated functional outcomes. Such a strategy aims to achieve precise regulation of protein stability and function.

Overall, it is widely acknowledged that palmitoylation modifications are correlated with in neurological disorders. Current, more studies are primarily focused on investigating the connection between AD and palmitoylation modifications. Nevertheless, additional exploration is required to clarify the specific role of palmitoylation in the pathogenesis of AD. To date, there is a limited understanding of the role of palmitoylation in neurological disorders. However, studies have consistently demonstrated the dysregulated levels of palmitoylation in these disorders. It is worth noting that the majority of studies on palmitoylation therapeutics are still in their infancy, and more in‐depth studies are needed to explore the clinical implications of palmitoylation modifications for neurological disorders.

## AUTHOR CONTRIBUTIONS

Yan‐Ran Qian drafted the manuscript, Yu‐Jia Zhao designed figures and tables, Feng Zhang revised the manuscript. All authors approved this manuscript for publication.

## CONFLICT OF INTEREST STATEMENT

The authors declared no conflicts of interest.

### ETHICS STATEMENT

Not applicable.

## Data Availability

Not applicable.
